# FAIR principles and the IEDB: short-term improvements and a long-term vision of OBO-foundry mediated machine-actionable interoperability

**DOI:** 10.1093/database/bax105

**Published:** 2018-02-19

**Authors:** Randi Vita, James A Overton, Christopher J Mungall, Alessandro Sette, Bjoern Peters

**Affiliations:** 1 La Jolla Institute for Allergy and Immunology, Division of Vaccine Discovery and Center for Emerging Diseases and Biodefense, 9420 Athena Circle, La Jolla, CA 92037, USA; 2 Lawrence Berkeley National Laboratory, Division of Environmental Genomics and Systems Biology, 1 Cyclotron Rd Berkeley, CA 94720, USA

## Abstract

The Immune Epitope Database (IEDB), at www.iedb.org, has the mission to make published experimental data relating to the recognition of immune epitopes easily available to the scientific public. By presenting curated data in a searchable database, we have liberated it from the tables and figures of journal articles, making it more accessible and usable by immunologists. Recently, the principles of Findability, Accessibility, Interoperability and Reusability have been formulated as goals that data repositories should meet to enhance the usefulness of their data holdings. We here examine how the IEDB complies with these principles and identify broad areas of success, but also areas for improvement. We describe short-term improvements to the IEDB that are being implemented now, as well as a long-term vision of true ‘machine-actionable interoperability’, which we believe will require community agreement on standardization of knowledge representation that can be built on top of the shared use of ontologies.

## Introduction

Recently, the principles of Findability, Accessibility, Interoperability and Reusability **(**FAIR; Textbox 1**)** have been formulated as critical goals that data repositories should meet to enhance the usefulness of their data holdings (1). These principles were established by a diverse group of academic and private community members with a particular emphasis on enhancing the ability of machines to automatically find and use data (Textbox 2). The authors of the FAIR data principles call on all data producers and publishers to examine and implement these principles and there is good indication that funding agencies and journals will increasingly require adherence to such principles.

**Textbox 1: The FAIR principles as stated inthe study by** Wilkinson *et al.***(**1)

**To be Findable:**

 F1. (meta)data are assigned a globally unique and persistent identifier

 F2. data are described with rich metadata (defined by R1 below)

 F3. metadata clearly and explicitly include the identifier of the data it describes

 F4. (meta)data are registered or indexed in a searchable resource

**To be Accessible:**

 A1. (meta)data are retrievable by their identifier using a standardized communications protocol

 A1.1 the protocol is open, free, and universally implementable

 A1.2 the protocol allows for an authentication and authorization procedure, where necessary

 A2. metadata are accessible, even when the data are no longer available

**To be Interoperable:**

 I1. (meta)data use a formal, accessible, shared, and broadly applicable language for knowledge representation.

 I2. (meta)data use vocabularies that follow FAIR principles

 I3. (meta)data include qualified references to other (meta)data

**To be Reusable:**

 R1. meta(data) are richly described with a plurality of accurate and relevant attributes

 R1.1. (meta)data are released with a clear and accessible data usage license

 R1.2. (meta)data are associated with detailed provenance

 R1.3. (meta)data meet domain-relevant community standards

**Textbox 2:** FAIR **goals for machine actionable data objects as stated inthe study by Wilkinson *et al.*(1)**

“The agent (…) [should] have the capacity, when faced with a digital object never encountered before, to (i) identify the type of object (with respect to both structure and intent); (ii) determine whether it is useful within the context of the agent’s current task by interrogating metadata and/or data elements; (iii) determine whetherit is usable, with respect to license, consent or other accessibility or use constraints; and (iv) take appropriate action, in much the same manner that a human would.”

**Figure 1.**
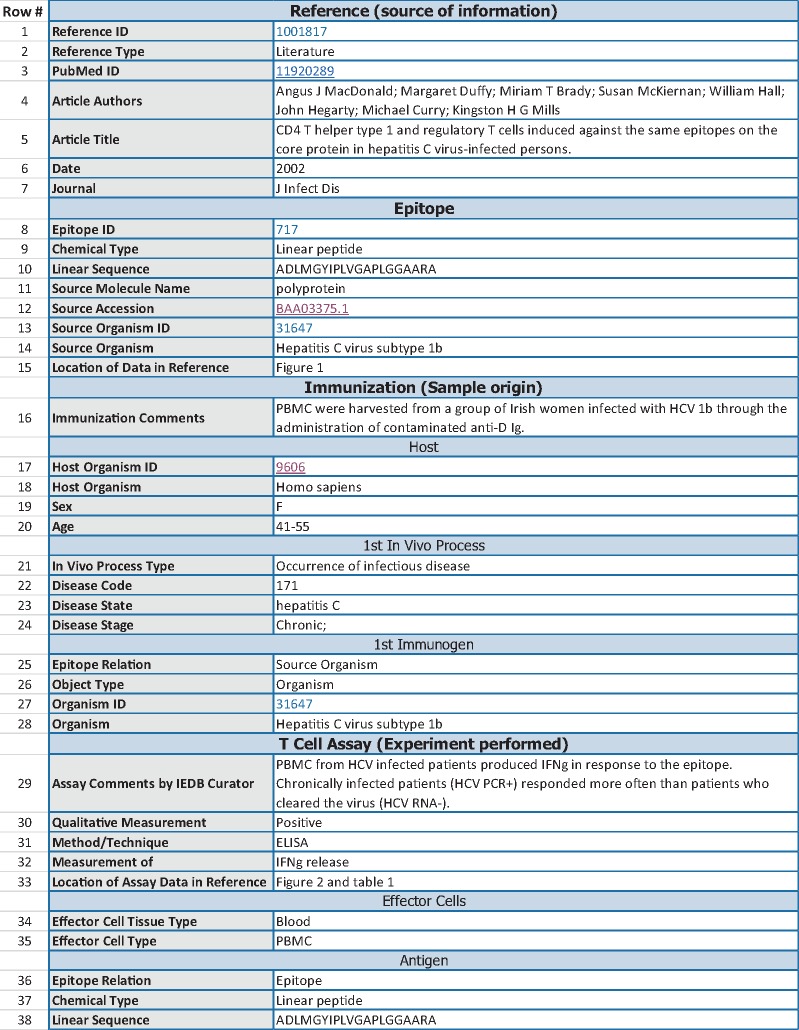
**Abbreviated IEDB record for one assay**. The data shown are an abbreviated version of the IEDB record for http://www.iedb.org/assay/1288921. The record is separated into sections (e.g. Reference, Epitope, Immunization), and subsections (e.g. Host within the Immunization section). The leftmost column contains a numeric ID for ease of reference in this manuscript that is not part of the IEDB. A representative subset of 38 of the original 63 data rows of the record are displayed, and some rows were re-ordered to improve readability of this table.

Following the example of other responsible databases such as UniProt (2), we wanted to assess how well the Immune Epitope Database (IEDB) (3) currently adheres to FAIR principles and how it could be further improved. In this context, it is important to note some characteristics of the IEDB in order to understand how it compares to other knowledge resources. The IEDB is a publically available database of experiments demonstrating recognition of immune epitopes by adaptive immune receptors. The IEDB was established in 2004 and provides access to over a million experiments manually curated from >18 500 journal articles, as well as data directly submitted by the community. The atomic unit of curation in the IEDB is an assay (i.e. an experiment) in which the immune recognition of an epitope is tested. Figure 1 gives an abbreviated example of data associated with one assay in the IEDB. The structured description of each experiment encompasses up to 400 database fields describing (i) the source of the information about the experiment (‘Reference’), (ii) the epitope structure itself (‘Epitope’), (iii) immune history of the host from whom samples are tested for recognition of the epitope (‘Immunization’) and (iv) the experimental techniques and type of immune response measured (‘Assay’). By structuring the free text information from the literature into the IEDB format, users can systematically query for experiments by many aspects of the data. For example, one can search by features of the host, such as experiments that were conducted using samples from Hepatitis C virus infected humans, as well as by features of what was measured, such as interferon gamma production by T cells in response to peptide epitopes.

The IEDB was designed primarily for human users querying through the website interface. Moreover, our user community predominantly consists of experimental scientists, so most of the effort to date has gone into making the query and reporting interfaces accessible without any advanced computational skills. We were thus expecting that the current IEDB implementation might not live up to the highest standards of the FAIR principles and especially concerning the machine action ability (Textbox 2) of our data. At the same time, throughout the last 10 year, significant effort has been made to make the IEDB data more computable. This was done to utilize automated inferences for data validation, to enable advanced query interfaces (4) and also to improve links from the IEDB to other knowledge repositories (5). Accordingly, we here conducted a review of the IEDB’s compliance with FAIR principles to provide a real life example of the degree of compliance of an existing resource, to identify areas of non-compliance and also to explore how the FAIR principles might be adjusted and fine-tuned to further improve their usefulness.

## Results

Several of the FAIR principles (e.g. F2 in Textbox 1) make a distinction between data and metadata. This distinction makes immediate sense for knowledge repositories that store raw data in a standardized format, such as sequence reads in FASTQ format in the Sequence Read Archive (SRA) (6) or Flow Cytometry Standard files in FlowRepository (7). Such ‘data’ must be accompanied with ‘metadata’ on how the data were generated. For example, in the case of an SRA deposition, information on what sample was being sequenced would be considered ‘metadata’, and specific sequence reads would be considered ‘data’. However, in the case of the information stored in the IEDB, there is no separation between data and metadata. Arguably, the IEDB stores only metadata, and the raw data can be found in the original journal article, typically in the form of figures or tables that follow no specific convention. In the following, we will thus interpret FAIR principles that explicitly refer to ‘data’ as applying to the information in the original journal articles, and to ‘metadata’ as the information stored in the IEDB database fields.

### Principle F: findable

There are four items (F1–F4, textbox 1) spelling out the principle of findability. We will address each of these items in the following paragraphs, starting each paragraph by repeating the principle as stated in Textbox 1 in *italics* and an assessment how the IEDB currently adheres to the principle and, if applicable, how the IEDB can be improved.

#### F1: (meta)data are assigned a globally unique and persistent identifier

The IEDB assigns unique identifiers, with the most fundamental being the identifier for a specific assay. For example, URL http://www.iedb.org/assay/1288921 identifies the IEDB record for the experiment displayed in Figure 1. In addition, a collection of assays curated from a single reference has a separate identifier. For example, http://www.iedb.org/reference/1001817 identifies the set of 34 assays curated from the journal article that included the experiment in Figure 1. By utilizing the full uniform resource locator (URL) as the identifier, we ensure that the identifier is globally unique, and that someone with an identifier can find more information about the resource using a web browser or other common tools. CURIE syntax can be used to define a mapping from ‘IEDB_ref’ to the URL http://www/iedb.org/reference/, allowing two-way translation between the CURIE ‘IEDB_ref: 1001817’ and the full URL http://www.iedb.org/reference/1001817. This gives us all the benefits of a compact ID and a findable URL. In terms of persistence, the IEDB is committed to retain these identifiers, and if there are changes to the identifier scheme, to utilize HTTP redirects to ensure that the URLs will continue to resolve. While there are dependencies on the continued control of the iedb.org domain name for long-term persistence, we believe that scenarios in which the IEDB continues to be available, but the domain does not are highly unlikely. Thus, we believe that the IEDB does follow the principles articulated in F1.

#### F2: data are described with rich metadata (defined by R1 below)

As explained earlier, the IEDB is a metadata repository, so striving to meet principle F2 is at the heart of its mission. How metadata is chosen is described in our response to R1 below.

#### F3: metadata clearly and explicitly include the identifier of the data it describes

The IEDB identifies the data being described by linking to the relevant journal publication in terms of citation information (journal, author, title, year, volume, pages, etc.) and more importantly for machine readable linkage, by the PubMed ID. In addition, the specific location in the journal article in which the curated information for the specific epitope and assay was found is described in separate database fields (Rows #15 and 33 in Figure 1). However, encoding the location in the journal article is currently done in free text. An improvement that is currently underway is to standardize the content of this field with the ultimate goal of using a structured vocabulary such as an ontology to describe locations in the journal article or possibly using anchor tags in the text or xml.

The data shown are an abbreviated version of the IEDB record for http://www.iedb.org/assay/1288921. The record is separated into sections (e.g. Reference, Epitope and Immunization) and subsections (e.g. Host within the Immunization section). The leftmost column contains a numeric ID for ease of reference in this manuscript that is not part of the IEDB. A representative subset of 38 of the original 63 data rows of the record are displayed, and some rows were re-ordered to improve readability of this table.

#### F4: (meta)data are registered or indexed in a searchable resource

The IEDB itself is an indexed, searchable resource of IEDB records. However, this principle is presumably intended for users (or machines) that are not aware that the IEDB exists, which necessitates discoverability of the IEDB data without knowledge of the IEDB itself. For our targeted user community of immunologists, we have performed user surveys, interviews and inspection of IEDB website access logs which identified that the main ways that our users become aware of the IEDB are (i) being pointed directly to the IEDB by reading a journal article mentioning it, (ii) general web-search engines, such as Google and (iii) links from immunology related knowledge repositories, such as the related Bioinformatics Resource Centers (BRC) (8) or the National Center for Biotechnology Information (NCBI) taxonomy (9). In terms of the FAIR goal of being findable by machines, only item (iii) of interlinking with related knowledge resources is helpful. To improve upon this, we have indexed IEDB data in Biosharing (10) and have submitted IEDB data to two websites specifically intended for machine discoverability, namely bioCADDIE (11) and Wikidata (12).

### Principle A: Accessible

There are two main items for the principle of accessibility, A1 and A2. A1 is further subdivided into two parts: ‘A1. (meta)data are retrievable by their identifier using a standardized communications protocol’, ‘A1.1 the protocol is open, free, and universally implementable’, ‘A1.2 the protocol allows for an authentication and authorization procedure, where necessary’. The main protocol used to obtain data from the IEDB is simply through HTTP. No authentication is required to access IEDB data. While this means that the IEDB satisfies this FAIR principle to the letter, it does not do so in regards to the data being optimally machine actionable as defined in Textbox 2: Implementing machine access to IEDB data over the HTML interface intended for human users would require ‘screen scraping’ the information from the IEDB web pages, which is inherently error-prone and unstable. There are alternatives to access IEDB data, namely to (i) export a set of query results in spreadsheet format and to (ii) download the entirety of the IEDB either in XML format or as a SQL database. While these alternative formats are more machine readable, an automated agent inspecting an IEDB data record as identified in Figure 1 would not be aware that alternative representations are available. Thus, a representation of the IEDB assay records that is better suited for machine readability should be provided, as outlined in the **Long-term vision section** of the discussion. We have begun adding machine-readable metadata to our IEDB web pages, beginning with provenance data. The metadata is encoded in JSON-LD format (13), following Google’s structured data recommendations, and can be easily translated into other concrete Resource Description Framework (RDF) formats. We plan to build on this incrementally, including more of the human-readable content of our web pages in machine-readable format.

The second principle for accessibility is A2. ‘metadata are accessible, even when the data are no longer available’. Given that the data referred to in the IEDB is from published journal articles, we have not yet experienced cases where the data source was no longer available. Even in the case of a retracted journal article, a copy of said article would still be available, but marked as retracted (or redacted) by PubMed. The metadata captured in the IEDB would remain available in either case. To date, we have not annotated these records as belonging to a retracted article, but will begin doing so in the near future.

### Principle I: Interoperable

#### I1: (meta)data use a formal, accessible, shared, and broadly applicable language for knowledge representation

The IEDB uses standard database tables which essentially consist of key: value pairs to represent its information. There is implicit semantic meaning of how the different values in an IEDB record interrelate. This typically poses less problems for humans, as contextual clues will, e.g. make it obvious that fields such as disease code, disease state and disease stage (Figure 1, rows 22–24) are interrelated. But a machine would not be able to reliably make this inference. To address this issue, an explicit representation of this information using RDF/OWL is under development, as discussed is the Long-term vision section of the discussion.

#### I2: (meta)data use vocabularies that follow FAIR principles

Whenever possible, the IEDB utilizes externally developed vocabularies to describe a given domain, primarily through the use of open biomedical ontologies (OBO) Foundry ontologies (14). The principles and practices of the OBO Foundry ensure that member ontologies are findable through the OBO registry, accessible through standardized interfaces, interoperable through the common use of the OWL standard and reproducible through the persistent availability of versioned copies of ontologies over time. If an existing OBO ontology in a domain does not provide a term needed by the IEDB, we submit new term requests. This improves both the IEDB data and enriches the ontologies we utilize. For example, the IEDB has contributed  >300 terms to ontology for biomedical investigations (OBI) (15),  >200 terms to the disease ontology (16), and >2400 terms to Chemical Entities of Biological Interest (17), among others. There are, however, several areas where currently no standards are available to represent data needed by the IEDB, either within the OBO Foundry or outside of it. In some cases, such as with major histocompatibility complex (MHC) restriction, the IEDB team developed its own ontology, as in the MHC restriction ontology (18).

There are a number of shortcomings in the way vocabularies are used by the IEDB. First of all, the use of external vocabularies is not consistently indicated to external users. For example, row 31 in which the method/technique of the experiment is specified as ‘ELISA’ refers to a class defined in OBI (http://purl.obolibrary.org/obo/OBI_0000661) and the value for what is being measured in row 32 (‘IFNg release’) is referring to a term in the gene ontology (http://purl.obolibrary.org/obo/GO_0032609). This must be made explicit to external users in a consistent fashion, so that every ‘value’ in a key-value pair has both a textual value and a URL resolving to a machine readable term, ideally from externally defined vocabularies. We will implement a consistent way of communicating where linkable identifiers refer to specific terms. We are considering utilizing RDFa to add attribute-level extensions to our HTML in the web pages in order to convey that the term on the page is from a formal ontology and comes with its own rich metadata.

Second, for a limited number of IEDB fields, no external vocabularies have been identified. In these cases, the IEDB uses list values that are not externally accessible and therefore do not comply with FAIR principles. In several instances, we anticipate the emergence of useful annotations, submitting our data to external groups ahead of the existence of stable identifiers, such as with inbred laboratory organisms that NCBI taxonomy does not cover. For example, we are awaiting stable identifiers for rat strains from the Rat Genome database (19) and for mouse strains from Mouse Genome Informatics (20). We have submitted our list of needs to both groups and will be ready to incorporate their official identifiers as soon as they are released. We are also currently working with the iPTMnet (21) to obtain formal identifiers for post-translational modifications (PTMs) of proteins and are always open to opportunities to better identify and annotate proteins found in our data. While we continue to rely on external vocabularies, in the cases when no external vocabularies have been identified, we strive to clarify their meaning and make the IEDB internal definitions of these terms explicit. Definitions for these terms are provided to the public in the IEDB curation manual (22) but could be made more explicit. We have also recently made these potentially temporary terms available as part of the ontology for immune epitopes (ONTIE) (23).

#### I3: (meta)data include qualified references to other (meta)data

The OBO family of ontologies makes a large number of semantically explicit cross-references to other ontologies. Uusing OBO ontology terms in its metadata annotation, the IEDB connects to this rich network of metadata.

### Principle R: Reusable

#### R1: meta(data) are richly described with a plurality of accurate and relevant attributes

The IEDB metadata representation is extremely detailed, describing individual experiments with up to 400 attributes. The data shown in Figure 1 are a shortened example. The relevance and accuracy of the attributes are checked by both a manual review process as part of our curation pipeline and automated validation checks integrated into the curation interface (22).

#### R1.1: (meta)data are released with a clear and accessible data usage license

The IEDB was failing in this principle when we began this analysis, as no license was specified. We have since addressed this shortcoming by including human readable licensing information for the Creative Commons Attribution 4.0 International (CC BY 4.0, https://creativecommons.org/licenses/by/4.0/) license and will be adding machine-readable licensing information, both at the website level and at the level of individual assay records shortly, using the ‘license’ predicate from the Dublin Core Metadata Initiative (24).

#### R1.2: (meta)data are associated with detailed provenance

There are two aspects of provenance to the IEDB metadata. First is the original data source that was curated by the IEDB, which is specified using PubMed identifiers and locations within the journal article as described in F3. In addition, the IEDB should state that the information entered as metadata for a given publication was authored by an IEDB curator. This was implied but not explicitly stated. We added this additional provenance information to the IEDB assay and reference web pages using the provenance authoring and versioning ontology (25), embedding it as machine-readable data in JSON-LD format, following Google’s structured data guidelines.

#### R1.3: (meta)data meet domain-relevant community standards

The domain of the IEDB is immunological investigations of epitope reactivity. There were no formal standards established in this specific area, so the design, implementation, and curation guidelines of the IEDB were instead vetted by the scientific immunology community through interactions with domain experts, publications, and outreach activities, including conference booths, annual workshops, and user surveys. As stated earlier, elements of the IEDB are expressed using external metadata standards, such as the use of the NCBI taxonomy to describe organism species.

### IEDB modifications to enhance compliance with FAIR principles

The short-term improvements that the IEDB could implement to better comply with FAIR principles are summarized in Table 1. The Long-term vision of how a truly machine-actionable data repository could be implemented, which will require extensive additional prototyping and coordination with other knowledge resource providers are detailed in the ‘Discussion’ section.

**Table 1.** Planned improvements of the IEDB to better adhere to FAIR standards

FAIR principle	Steps to improve IEDB compliance	Completion Date
F3	Standardize identification of journal parts (figures/tables)	10/2017
F4	Add IEDB metadata to Biosharing, Biocaddie and Wikidata	10/2017
A1	Provide machine actionable representation of IEDB assay level data	?
I1	Represent the IEDB data in RDF/OWL	?
I2	Make all links to external vocabularies explicit	06/2017
I2	Make all internal vocabularies public via ONTIE and link to them	06/2017
R1.1	Include licensing information with the IEDB records	12/2016
R1.2	Include provenance information regarding IEDB curation	12/2016

As listed in Table 1, we have identified eight areas for improvement of the IEDB that we began implementing in 2016 and expect to complete by the middle of 2018:

### F3: Identification of journal figures and tables

- We identified patterns (e.g. ‘Figure X’) and common term composition rules (e.g. ‘Figure X and Table Y;, or ‘Figure X, Table Y’) for location of data in figures
- We developed a canonical representation and are transitioning data to this format
- We will implement validation rules enforcing a format that is convertible to canonical
- We will identify an ontology that covers journal article parts used in IEDB location fields
- We will add URLs to ontology in addition to free text

### F4: Add IEDB data to external resources

- IEDB has been indexed by Biosharing
- IEDB datasets have been sent to Biocaddie and modeling is in process
- Relevant IEDB links and datasets have been identified by Wikidata collaboration and their implementation is in progress

### I2: Make all links to external vocabularies explicit

- We will standardize the format of lookup tables
- We will consistently report values with textual label and link to external resources
- We have enabled exports with external identifiers

### I2: Make all internal vocabularies public via ONTIE and link to them

- We have identified all values in the IEDB tables not taken from external vocabularies
- We have assigned each an ONTIE identifier
- We will organize ONTIE terms in passable hierarchy
- We have registered ONTIE for PURLs

### R1.1: Include licensing information with the IEDB records

- We identified the Creative Commons Attribution 4.0 International License as appropriate for IEDB data
- Human readable licensing information has been added to the IEDB download and citation sections
- We will add human and machine readable licensing information to the IEDB assay and reference pages

### R1.2: Include provenance information regarding IEDB curation

- We added machine-readable provenance information in JSON-LD to the IEDB assay and reference pages

## Discussion

We systematically inspected the IEDB for adherence to the FAIR principles. Overall, the IEDB does comply with a number of the FAIR principles to a high standard, but at the same time, several areas for improvement were identified. Inspection of the IEDB for compliance to the well-argued FAIR principles enabled the IEDB team to take an outside viewpoint of the IEDB which helped to identify weaknesses that were not clearly apparent to those involved in the development and day-to-day operation of the database. Conversely, it was apparent to us that improvements could be made in the formulation of FAIR principles, to facilitate understanding of what specifically each principle was meant to address, and how each principle differentiates from the other principles. For example, a definition of what is considered ‘data’ vs. ‘metadata’ can be found in a related publication (26), but it is not clear if this is the official FAIR definition, or alternatively if this distinction could be altogether eliminated. Likewise, an additional document, distinct from the FAIR publication, clearly detailing each of the FAIR principles with examples of adherence and non-adherence would be key to facilitate widespread implementation and adherence to the principles. Additional documentation in existence on various web sites does not match the numbering or wording of the published principles, so it becomes paradoxically ambiguous what it means to ‘adhere to FAIR principles’ when using those secondary sources. Recent FAIR publications and use cases (2, 26, and 27) continue to clarify what FAIR really means and we expect that as more practical applications of FAIR principles are performed, increasingly more of the ambiguity of ‘FAIRness’ will be resolved. We hope that our interpretation of these principles will also contribute to the general understanding.

### Long-term vision

A truly machine-actionable data repository, in which an automated agent for the first time encountering the repository can inspect it and act like a human would (Textbox 2), will require substantial additional work beyond the implementation of FAIR principles. Specifically, even if two knowledge resources use the same syntactic representation (e.g. RDF/OWL) and utilize the same vocabularies (e.g. OBO Foundry ontologies), in our experience interoperability on a machine level will still be difficult to achieve. For example, because of limitations of OWL not being expressive enough, it is difficult to ensure that a computer understands how different modeling schemes refer to the same object. To achieve this goal will require establishing a set of conventions that can be validated to ensure that a machine will understand content modeled by different sets of people.

For example, if the PHI-base pathogen–host interaction database (28) extracts host: pathogen information and the IEDB contains the very same information, but models it in an incompatible way, an automated agent would not be able to determine that the information is in fact the same. Neither model is necessarily right or wrong, but they would need to be manually coordinated and resolved before automation would be possible.

We suggest that the effort involved in OBO Foundry ontology creation is also the best venue to provide guidance on how to model statements using OBO Foundry terms. We are in the process of actively coordinating the modeling of datasets in different NIAID funded repositories already committed to using OBO Foundry ontologies. Specifically, these include the BRCs (8), the IEDB and ImmPort (29). We plan to use this as a proof of principle on how to cross-integrate data from different ontologies towards the future goal of a truly machine-actionable data repository.

## Author contributions

BP conceived of and directed the overall study. RV, JAO and BP performed the detailed assessment of principles. JO guided the technical implementation. All authors contributed to the plans for addressing limitations in the IEDB and to the writing of the manuscript.

## Funding

Funding is provided by The National Institutes of Health (HHSN272201200010C and 1R24HG010032–01).

**Competing interests.** The authors declare no competing financial interests*.*

## References

[1] Wilkinson M.D., Dumontier M., Aalbersberg I.J. (2016) The FAIR Guiding Principles for scientific data management and stewardship. Sci. Data, 15; 3, 160018. 26978244 10.1038/sdata.2016.18 PMC4792175

[2] Inside UniProt, Being FAIR at UniProt. http://insideuniprot.blogspot.com.es/2016/11/being-fair-at-uniprot.html (30 October 2017, date last accessed).

[3] Vita R., Overton J.A., Greenbaum J.A. (2015) The immune epitope database (IEDB) 3.0. Nucl. Acids Res., 43, D405–D412. RRID: SCR_006604. 25300482 10.1093/nar/gku938 PMC4384014

[4] Vita R., Overton J.A., Greenbaum J.A. (2013) Query enhancement through the practical application of ontology: the IEDB and OBI. J. Biomed. Semantics, 15; 4, S6. 23734660 10.1186/2041-1480-4-S1-S6 PMC3633001

[5] Peters B., Sette A. (2007) Integrating epitope data into the emerging web of biomedical knowledge resources. Nat. Rev. Immunol., 7, 485–490. 17479127 10.1038/nri2092 PMC7097317

[6] Leinonen R., Sugawara H., Shumway M. (2011) The sequence read archive. International Nucleotide Sequence Database Collaboration. Nucl. Acids Res., 39, D19–D21. 21062823 10.1093/nar/gkq1019 PMC3013647

[7] Spidlen J., Breuer K., Rosenberg C. (2012) Flowrepository—a resource of annotated flow cytometry datasets associated with peer-reviewed publications. Cytometry, 81A, 727. 10.1002/cyto.a.22106 22887982

[8] https://www.niaid.nih.gov/research/bioinformatics-resource-centers (30 October 2017, date last accessed).

[9] NCBI Resource Coordinators. (2017) Database Resources of the National Center for Biotechnology Information. Nucl. Acids Res., 45(Database issue), D12–D17. 27899561 10.1093/nar/gkw1071 PMC5210554

[10] McQuilton P., Gonzalez-Beltran A., Rocca-Serra P. (2016) BioSharing: curated and crowd-sourced metadata standards, databases and data policies in the life sciences. Database (Oxford), 2016, baw075. 27189610 10.1093/database/baw075 PMC4869797

[11] Ohno-Machado L., Alter G., Fore I. (2015) bioCADDIE White Paper—Data Discovery Index. *Figshar*e 10.6084/m9.figshare.1362572.

[12] Burgstaller-Muehlbacher S., Waagmeester A., Mitraka E. (2016) Wikidata as a semantic framework for the Gene Wiki initiative. Database, 2016, 1–10. 10.1093/database/baw015 PMC4795929 26989148

[13] https://json-ld.org (30 October 2017, date last accessed).

[14] Smith B., Ashburner M., Rosse C. (2007) The OBO Foundry: coordinated evolution of ontologies to support biomedical data integration. Nat. Biotechnol., 25, 1251–1255. 17989687 10.1038/nbt1346 PMC2814061

[15] Bandrowski A., Brinkman R., Brochhausen M. (2016) The ontology for biomedical ivestigations. PLoS One, 11, e0154556. 27128319 10.1371/journal.pone.0154556 PMC4851331

[16] Kibbe W.A., Arz C., Felix V. (2015) Disease Ontology 2015 update: an expanded and updated database of human diseases for linking biomedical knowledge through disease data. Nucl. Acids Res., 43, D1071–D1078. 25348409 10.1093/nar/gku1011 PMC4383880

[17] Hastings J., de Matos P., Dekker A. (2013) The ChEBI reference database and ontology for biologically relevant chemistry: enhancements for 2013. Nucl. Acids Res., 41(Database issue), D456–D463. 23180789 10.1093/nar/gks1146 PMC3531142

[18] Vita R., Overton J.A., Seymour E. (2016) An ontology for major histocompatibility restriction. J. Biomed. Semantics, 7, 1. 26759709 10.1186/s13326-016-0045-5 PMC4709943

[19] Shimoyama M., De Pons J., Hayman G.T. (2015) The Rat Genome Database 2015: genomic, phenotypic and environmental variations and disease. Nucl. Acids Res., 43, D743–D750. 25355511 10.1093/nar/gku1026 PMC4383884

[20] Blake J.A., Eppig J.T., Kadin J.A. (2017) Mouse Genome Database (MGD)-2017: community knowledge resource for the laboratory mouse. Nucl. Acids Res., 45, D723–D729. 27899570 10.1093/nar/gkw1040 PMC5210536

[21] http://research.bioinformatics.udel.edu/iptmnet/ (30 October 2017, date last accessed).

[22] Vita R., Peters B., Sette A. (2008) The curation guidelines of the immune epitope database and analysis resource. Cytometry A, 73, 1066–1070. 18688821 10.1002/cyto.a.20585 PMC2597159

[23] Greenbaum J.A., Vita R., Zarebski L. (2009) Representing the Immune Epitope Database in OWL. In: *Proceedings of the 12th Annual Bio-Ontologies Meeting.* International Society for Computational Biology, Stockholm, Sweden, pp. 45–48.

[24] http://dublincore.org/ (30 October 2017, date last accessed).

[25] Ciccarese P., Soiland-Reyes S., Belhajjame K. (2013) PAV ontology: provenance, authoring and versioning. J. Biomed. Semantics, 4, 37. 24267948 10.1186/2041-1480-4-37 PMC4177195

[26] Wilkinson M.D., Verborgh R., da Silva Santos L.O.B. (2016) Interoperability and FAIRness through a novel combination of web technologies. Peer J., 4, e2522v1.27761324

[27] Mons B., Neylon C., Velterop J. (2017) Cloudy, increasingly FAIR; revisiting the FAIR Data guiding principles for the European Open Science Cloud. Inform. Services Use, 37, 49–56.

[28] Urban M., Pant R., Raghunath A. (2015) The Pathogen-Host Interactions database: additions and future developments. Nucl. Acids Res., 43, D645–D655. 25414340 10.1093/nar/gku1165 PMC4383963

[29] Bhattacharya S., Andorf S., Gomes L. (2014) ImmPort: disseminating data to the public for the future of immunology. Immunol. Res., 58, 234–239. 24791905 10.1007/s12026-014-8516-1

